# Association of Co-Living and Age on the Type of Sports Practiced by Older People

**DOI:** 10.3390/sports10120200

**Published:** 2022-12-05

**Authors:** María Antonia Parra-Rizo, Felipe Díaz-Toro, Fatine Hadrya, Patricia Pavón-León, Igor Cigarroa

**Affiliations:** 1 Faculty of Health Sciences, Valencian International University (VIU), 46002 Valencia, Spain; 2 Department of Health Psychology, Faculty of Social and Health Sciences, Campus of Elche, Miguel Hernandez University (UMH), 03202 Elche, Spain; 3 Departament of Epidemiology, Mailman School of Public Health, Columbia University, New York, NY 10032, USA; 4 Facultad de Enfermería, Universidad Andres Bello, Santiago 8370035, Chile; 5 Departament of Health Sciences, High Institute of Health Sciences, Hassan First University of Settat, Settat 26000, Morocco; 6 Laboratory of Biology and Health, Faculty of Sciences, Ibn Tofail University, Kenitra 14000, Morocco; 7 Health Sciences Institute, Universidad Veracruzana, Veracruz 91190, Mexico; 8 Escuela de Kinesiología, Facultad de Salud, Universidad Santo Tomás, Los Ángeles 4440000, Chile

**Keywords:** living alone, living in company, type of company, older people, elderly, physical activity, gymnastics, aquatic exercise, dance, yoga, pilates training

## Abstract

Introdution: The environment and the type of co-living of older people are crucial to understanding how the nature of their context influences a healthy lifestyle. However, no studies have investigated to what extent their type of co-living may be associated with the sports and the profile practice according to their age. Objective: This study aimed to assess the association between the types of co-living and the age of the physically active elderly and the sports they practice. Hypothesis: It is thought that the age, situation, and satisfaction with the way of living in physically active older people differ according to the type of sport they practice. Methods: Cross-sectional study. We included 358 individuals aged between 61 and 93 years old (M = 69.66, SD = 4.74). Type of co-living was classified as living alone or living with others. The sports activities evaluated were: gym, dance, water activities, and yoga/pilates. Differences in the type of co-living and sports practiced were evaluated by ANOVA or Chi2. Results: Among the elderly who practice gymnastics, most of them live alone and in a tight core (*p* < 0.001) (*Phi* = 0.244). Furthermore, those who practice aquatic activities are more frequently the youngest (*p* < 0.001) (*Phi* = 0.198). Conclusion: Older people who do gymnastics have smaller living groups, those under 69 opt for gymnastics and aquatic activities, while those aged 70 and over prefer dance, yoga, and pilates.

## 1. Introduction

The existing scientific literature at international level recommends physical activity in older adults as a preventive measure of functional and cognitive deterioration [1]. As is well known, aging leads to an increase in the prevalence of chronic diseases and it has a strong impact on socio-health costs. Therefore, physical activity is essential at this vital stage since it positively affects the quality of life of older people; it prevents the deterioration of their motor functions and the possible falls associated with disability [2]. The practice of physical activity also prevents sarcopenia, muscle loss [3,4], as well as cardiovascular problems, both through resistance exercise and aerobic exercise [5]. Conversely, little physical activity or sedentary behavior leads to a greater predisposition to developing somatic and mental disorders. In contrast, physical training and sport are associated with better mental health and, therefore, have a preventive value in developing depression, dementia, and cognitive impairment [6]. Other authors [7,8] found that a high level of physical activity was related to greater satisfaction with life, subjective well-being, better functional skills, fewer difficulties in carrying out activities of daily living, and a higher assessment of their autonomy and health. There are relevant variables that influence practice, such as some socio-contextual elements linked to quality of life, a high level of education and high income as facilitating elements, being a woman, having less functional capacity, or being less involved in leisure activities as barriers to encouraging the practice of physical activity.

Regarding the benefits of each sport modality in the elderly, this matter has been extensively studied, although without linking it to the type of co-living they maintain. For instance, dancing may reduce cognitive deterioration [9] and allows cognitive and physical abilities to be maintained, improving the quality of life of older people [10]. On the other hand, a study with pre-frail older women who practiced aquatic exercise found improvements in tension-anxiety, depression and confusion, and anger-hostility [11]. Further investigations found improvements in body composition, functional aptitude, and cognitive function in 102 older women [12]. Likewise, an aquatic exercise program lasting 12 weeks (twice a week) found improvements in depression, anxiety, sleep, and functional autonomy [13].

Furthermore, gymnastics reduces the risk of suffering falls at advanced ages [14] and contributes to successful aging and the maintenance of a good social life [15].

Likewise, yoga has been shown to produce improvements in muscle strength, balance, mobility, and flexibility in older people [16]. It also reduces anger, anxiety, improves self-efficacy, well-being, executive function [17], and sleep [18]. Moreover, practicing pilates improves balance and strength. In a study conducted with a group of 46 elderly people, it was observed that pilates had greater effects than a general physical activity program and that its practice resulted in improved postural control in the participants [19]. Other research on the effects of pilates on the elderly has found improvements in balance as well as lower risk of falls in women older than 60 years [20]; in physical functioning and quality of life in women with osteoporosis [21]; in mental and cognitive function, anxiety and depression, with an increase in VO2 (maximum volume of oxygen), a decrease in BMI (Body Mass Index) and a drop in daytime salivary cortisol in 27 healthy older women between the ages of 60 and 65 years [22]; in autonomy in activities of daily living, balance, strength, and flexibility in people over 60 years of age [23]; and in the improvement of depression in a group of 75 older women [24].

Among the most important strategies for disease prevention and health promotion on a physical and cognitive level, along side physical exercise, are health, social relations, and staying active [25]. In this regard, a study carried out on a European level with a sample of 68,844 people over the age of 50, found a prevalence of multimorbidity (presence of two or more health conditions) in 28.2% of men and 34.5% of women. The independent variables were gender, age group, educational level, self-perceived health, loneliness, network size, quality of life, Body Mass Index (BMI), and disability. The most common health conditions were cardiometabolic and osteoarticular diseases in both genders, and emotional disorders in younger women. Multimorbidity was associated with sociodemographic and physical characteristics, self-rated health, quality of life, and loneliness [26]. This latest finding suggests that a relevant aspect of quality of life is the social factor, that is, the one referring to the type of co-living practiced by the elderly; if they live alone or with company, as well as the relationships with their health. Research carried out with a sample of 568 adults over 65 years of age who lived alone, found an increase in depressive symptomatology [27] and an increased risk of mortality [28] among those living alone. This isolation was also associated with worsening health and psychological state, among those with low and middle income [29]. Another author found that loneliness, isolation, or living alone affects health and is negatively associated with psychological well-being in 3553 older people, being necessary for promoting social support and friendly environments to reduce such loneliness and isolation [30]. In the same way, other researchers [31] also found that social factors and the number of household members affected health and well-being. In this regard, a study carried out with 4772 adults over the age of 65 who lived alone, showed that older people were at risk of poor health, and this was attenuated if they received social support, which was also associated with a lower risk of health care and shorter admission time [32]. Likewise, in an investigation carried out with 314 people between 70 and 84 years of age, it was observed that the fact of living alone was associated with poor mental health, while leisure activities could moderate this issue. Therefore, it may be deduced that interventions should be aimed at promoting participation in leisure activities [33]. Other authors relate isolation to depression and the falls that occur in old age [34] and conclude that social environment, family relationships, and social connection are essential factors for successful aging [35].

Another study, carried out with 7759 people aged between 65 and 84, showed that exercising improved physical and mental function alone or in a company. However, if the practice was done in a company, it improved physical and mental function [36].

The scientific literature on this topic addresses the relationship between being alone, living alone, and loneliness with physical and/or psychological health issues, and studies the health benefits that practicing sport has for older people. However, the research on the subject does not deal with the differences according to the type of co-living or age in the kind of sports practiced, nor the kind of exercise done (gymnastics, dance, yoga, pilates, or aquatic activities). Hence, the novelty of this study aimed at discovering the differences in the co-living situation of older people and how the type of sports practiced differs depending on the age range.

The aim of this work is to study whether the participants’ age, situation, and satisfaction with their way of living together differ according to the type of physical activity they practice. It was hypothesized that their age, situation, and satisfaction with their way of living together vary according to the type of sport.

## 2. Method

### 2.1. Participants and Procedure

In this study, 358 older adults living in Elche (Alicante) were included. At present, the older population represents 19.77% of the population in Spain, a large representative percentage that will continue to grow in the coming years compared to children and young people [37]. The sample is non-probabilistic of incidental type.

Inclusion criteria were set as (1) being over 60 years old; (2) being physically active; (3) being physically active for at least one year; (4) practicing one sporting activity; (5) activities in a group environment. The exclusion criteria were: (1) limitations in reading and answering the questionnaires.

This is a cross-sectional study. The participants were selected in various contexts: sports centers, social centers, and outdoor spaces for sports practice.

On the one hand, 38 manager centers were contacted, 18 of them agreed to collaborate. The attendees interested in participating were given the informed consent and the questionnaire that they completed individually after practicing physical activity.

On the other hand, in outdoor sports practice spaces, contact was established with older people who met the defined requirements. Those who were interested in participating were given an envelope with the informed consent and the questionnaire that they had to return completed at a later appointment. In the mentioned questionnaire, the participants answered several questions about the type of sport they regularly practiced, they indicated what type of sport they practiced, the co-living they had, their satisfaction with it, and certain sociodemographic variables. This study was approved by the Ethics Committee of the Miguel Hernández University of Elche, Spain (200115191342) and the principles of the Helsinki Declaration of Ethics were followed.

### 2.2. Variables and Instruments

#### 2.2.1. Brief Quality of Life Questionnaire, CUBRECAVI

For the evaluation of satisfaction with the type of co-living, a CUBRECAVI quality of life questionnaire was used. This instrument is based on the multidimensional concept of quality of life and health proposed by the WHO. This self-administered questionnaire evaluates the most relevant components of quality of life in older people. It is made up of 21 subscales, among which the satisfaction with co-living used for this publication stands out. The participants must evaluate their degree of satisfaction on a Likert-type response scale with respect to items such as “*indicates the degree of satisfaction with co-living*”, where 0 is dissatisfied, 1 is indifferent, and 2 is satisfied. This questionnaire has a level of internal consistency of the scales (Cronbach’s alpha) that ranges between 0.70 and 0.92 [38]. The duration of the questionnaire is approximately 20 min and has recently been used in older people [39]. It is a highly recommended questionnaire to assess quality of life [38].

#### 2.2.2. Sociodemographic Questionnaire

Self-made instrument composed of 16 items related to gender, age, marital status, social and family environment, health status, type of co-living, life habits of the participants, and types of sports practiced. The scales of measurement used in defining the items were nominal, ordinal, and scaled, depending on the subject matter.

### 2.3. Analysis of Data

To analyze the differences between the participants according to the sport they practiced (gym, dance, water activities, and yoga/pilates). One-factor analysis of variance was applied to the quantitative variables and the chi-square test for qualitative variables.

The size of the effect was estimated using *ƞ*^2^ in the ANOVAs and, for qualitative variables, Phi Coefficient and Cramer’s V (according to the size of the contingency tables) were employed.

The significance value established was <0.05.

The data analyses were performed with the SPSS statistical package, version 23.0. (IBM Corp., Armonk, NY, USA for Windows).

## 3. Results

### 3.1. Co-Living Situation

The minimum age was 61 years and the maximum was 93, with a mean age of 69.66 years (SD = 4.74). A total of 64% were women and 36% were men. Regarding their marital status, 67.6% were married, 16.8% were widowed, 9.1% were single, and 6.5% were divorced. A total of 32.5% of the participants lived alone.

Concerning their health-related habits, 91.8% were non-smokers and 51.9% never drank alcohol. With reference to their state of health, 72.6% had no physical ailments and 88% had no psychological illness.

A total of 46.1% of the participants were highly physically active, 41.6% were moderately physically active, and 12.3% were moderately physically active. The types of physical activities practiced were: 54.7% gymnastics, 16.2% yoga/pilates, 15.1% dance activities, and 14% aquatic activities. A total of 32.5% of the participants lived alone, and 67.5% lived with other people. Of those living alone, 42.9% practiced gymnastics, 25.9% dance, 16% water activities, and 17.5% yoga/pilates. On the other hand, among those living with more people, 57.1% practiced gymnastics, 74.1% dance, 84% water activities, and 82.5% yoga/pilates.

Statistically significant differences in the structures of co-living according to the physical activity were found (*χ*^2^(3, *N* = 357) = 22.668; *p* < 0.001; *V_Cramer_* = 0.252) detecting the differences between the participants who do gymnastics and the others (42.9% live alone versus 19.9%) (*χ*^2^(1, *N* = 357) = 21.283; *p* < 0.001, *Phi* = 0.244). Table 1 shows the types of co-living that people reported.

The number of people with whom the participants live (Table 2) has been studied and from its analysis, it is observed that there are differences between the average number of people who live with the elderly depending on the type of sport practiced (*F*(3, 353) = 11,790, *p* < 0.001, *ƞ*^2^ = 0.091); specifically, the differences are recorded among the participants who do gymnastics (they live with an average of 1.02 people), the participants who do dance activities (1.59) (*p =* 0.010), the participants who do aquatic activities (1.98) (*p <* 0.001) and those who practice yoga/pilates (1.65) (*p =* 0.003).

Given that, as reflected in Table 3, only one participant says he/she is dissatisfied with his/her situation of co-living, specifically it has been analyzed if there are significant differences between those who declare themselves satisfied with their co-living and between those who are indifferent (99.7% of the sample) and the results indicate that there are no differences in the level of satisfaction with their situation of co-living manifested by the participants according to the sport they practice (*χ*^2^(3, *N* = 348) = 2.361; *p =* 0.501; *V_Cramer_* = 0.082

### 3.2. Age

The analysis related to age (Table 4) has revealed that there are statistically significant differences in the age groups (segmented according to the median) in which the participants are included according to the physical activity they practice (*χ*^2^(3, *N* = 358) = 14.146; *p =* 0.003; *V_Cramer_* = 0.199).

Reviewing in which physical activities age differs, it has been found that the differences are between those who do gymnastics/water activities with those who practice dance/yoga/pilates (up to 69 years old they are 73.6% compared to 53.6%) (*χ*^2^(1, *N* = 358) = 14.001; *p* < 0.001; *Phi* = 0.198).

## 4. Discussion

The objective of this research was to study the association between the type of co-living, the age of the participants, and the type of sport they practice.

In the first place, our results indicate differences in the participants’ co-living situations: among those who live alone, most of them practice gymnastics. In this regard, it should be noted that there are no studies that address the effect that co-living has on the types of sports practiced. However, in a study carried out with people aged 60 to 74 years, it was found that the majority of those who did not perform any physical-sports activity had a negative culture towards the practice of sports and also practiced low-intensity sports [40]. Another study of 7759 people aged from 65 to 84 found that compared to not exercising, exercising alone or with others was much better for good physical and mental function; however, in comparison to this study, our findings show differences according to the different sport modalities and their type of company. In fact, it is necessary to promote healthy aging, taking measures to promote exercise, well-being, and general quality of life, as well as to prolong healthy longevity [41].

This research, however, showed that physical exercise practiced with company improved both physical and mental function [17]. Additionally, older adults who live alone and are inactive are more likely to experience frailty or disability due to decreased physical activity. It must be taken into account that the study was carried out in a COVID-19 context [42]. In addition, studies focus on increasing the levels of physical practice and are excessively focused on the benefits that this has for health. It is, therefore, necessary to broaden the goals and aspirations in the study of the elderly, especially regarding satisfaction and increased participation in physical activities. In this sense, it is a priority to foster collective group activities and to create daily routines that can help to overcome the real barriers contributing, in this way, to achieve a fulfilling old age.

Nevertheless, in this population the diseases decrease the level of physical activity. The place of residence, for example, is vital: those who live in rural areas have a higher physical fitness than those who live in residences or in urban areas. Advanced age; low educational level; the habit of smoking; the presence of hypertension; musculoskeletal disorders, etc., are also decisive aspects for good aging [43].

However, despite the relevance of these results, there are no investigations that deal with the effect of the company in the elderly depending on the sport modality that they practice. Therefore, involving family and/or friends in sports practice seems to increase participation and social relationships in the environment in sports such as gymnastics and water activities.

Secondly, our results do not show any difference in satisfaction with co-living expressed by the elderly according to the type of sport they do. In this regard, only a couple of investigations have been found that address this topic [7,8]; hence, the novelty of our study. Nevertheless, studies adjacent to or close to our topic indicate an increase in life satisfaction in those with high or moderate levels of physical activity [44].

Thirdly, our results indicate differences in the age of the elderly according to the sport they perform. Research dealing with the influence of the type of co-living (or loneliness) and the choice of sport practiced by older people is also non-existent, which highlights, once again, the need to develop research that handles these variables. As can be seen, our results bring new knowledge to an area that needs to be investigated for health reasons. In fact, recently in an experimental study, they observed that walking had a significant effect on blood pressure and blood glucose [45]. Similarly, after physical training in a case of post-stroke dementia, improvements were observed in regaining strength, cognitive function, attention, and memory [46], and even against sarcopenia as a disorder of aging associated with many musculoskeletal and nervous system problems, attenuating muscle reduction, improving body instability, falls and injuries, so active activities and active lifestyles can delay the process and avoid instability due to lack of muscle, lack of coordination and injuries [47]. In addition, apart from all the benefits that it brings, in itself, indirectly due to its practice, being in good health and having functional capacities that allow basic activities of daily living to be performed, are predictors of good satisfaction with life [48].

As theoretical implications, this work represents an advance in the study of healthy lifestyle habits and, in particular, sports practice; as well as the importance of age in some sports activities. It is also necessary to delve into the limitations in the practice of any sports modality beyond 70 years if active aging is to be promoted, as well as the influence of the closest environment as an agent of social health that promotes greater sports practice. Therefore, as practical implications, it could be highlighted that this work can help improve intervention programs aimed at people over 70 years of age. Likewise, understanding the extent to which co-living can affect the type of sport practiced by the elderly person is relevant for correctly planning health interventions and designing innovative programs related to sport, the type of company, and the acquisition of healthy habits; since it has been seen that sports practice in the company of a family member from the environment of habitual co-living is a relevant factor.

Regarding the limitations of this research, the sample size is referred to, as well as its characteristics. That is to say, the participants are older people who are physically active, which does not allow extrapolating these results to other types of older people, outside the field of the physically active. This fact, nevertheless, is a strong point of the study, as it is very difficult to achieve a sample of older people with these characteristics. In the same way, when considering the limitations, it should be kept in mind that the study does not homogeneously cover the entire range of advanced ages, and that greater participation of women than men could affect the generalization of the results. Likewise, the fact of participating or having a family and, in the exclusion criteria, any comorbidities that the participants may have. The location in which participants have been recruited might have biased the results in terms of the sports they participated in and the living situation, such as sports centers and social centers, since the sports they participated in were likely to be highly dependent on the activities offered by these centers. The location of these centers could have been situated in areas with a higher density of older people living alone, for example. These are the limitations to be taken into account for future research.

Regarding the instruments used, it would be advisable to use biometric devices to evaluate physical activity. However, one of the strengths of this publication is that it addresses new variables such as living together at home, the differences by age range, and the types of sports.

Taking these data into account, a future line of research could be to study the sports profiles of the elderly and the relationships in their physical and emotional health from their social and family environment, with the aim of knowing the influences that these aspects have on the behavior and psychophysical health beyond 70 years. The variable of gender and its effect and/or state of health could be also considered.

## 5. Conclusions

The study showed that older people who practice gymnastics have smaller groups of co-living, but, despite this, they are equally satisfied as those who carry out other activities. Likewise, the age of the participants differs between each sport, with those under 69 years of age choosing to do gymnastics and aquatic activities, compared to those over 70 years of age who prefer dance, yoga, and pilates. In addition, the structure of co-living seems to affect the choice of sports, depending on whether they live alone or with more people in the household. Finally, we will highlight that these issues must be addressed when planning active aging policies, as well as non-pharmacological preventive health measures. The ultimate goal is to promote sports activities in order to guarantee psychophysical health and quality of life in octogenarians and nonagenarians.

## Figures and Tables

**Table 1:** Frequencies of the types of co-living according to the sport practiced *.

	Living Alone		With More People		*χ* ^2^
	*n*	*%*	*n*	*%*	
Gym	84	42.9	112	57.1	22.668 **
Dance	14	25.9	40	74.1	
Water activities	8	16.0	42	84.0	
Yoga/Pilates	10	17.5	47	82.5	

Note: *n* = number of participants; % = percentage; * *N* = 357; there is one participant whose type of co-living is unknown; ** *p* < 0.05.

**Table 2:** Means, standard deviations, and ANOVA of a factor of the number of people with whom the participants live according to the sport they practice.

	Gym		Dance		Water Activities		Yoga/Pilates				
	*n*	*M* *(SD)*	*n*	*M* *(SD)*	*n*	*M* *(SD)*	*n*	*M* *(SD)*	*F*	*p*	*ƞ* ^2^
Number of people the participants lived with	196	1.02 (1.11)	54	1.59 (1.33)	50	1.98 (1.33)	57	1.65 (1.14)	11.790	0.000 *	0.091

Note * *p* < 0.05; *n* = number of participants; *M* = Mean; *SD* = Standard deviation *F* = *F* statistic; *p* = *p*-value; *ƞ*^2^ = size of the squared eta effect.

**Table 3:** Frequencies in the subscale of satisfaction with co-living of social integration (CUBRECAVI) according to the sport practiced *.

	Dissatisfied		Indifferent		Satisfied		*χ* ^2^
	*n*	*%*	*n*	*%*	*n*	*%*	
Gym	0	0.0	19	9.7	177	90.3	2.361 **
Dance	1	2.0	7	14.3	41	83.7	
Water activities	0	0.0	8	16.0	42	84.0	
Yoga/Pilates	0	0.0	5	9.3	49	90.7	

Note: *n* = number of participants; % = percentage; * *N* = 349; there are nine participants that have not expressed their satisfaction with their type of co-living; ** *p* < 0.05.

**Table 4:** Frequencies of the age intervals according to the sport practiced *.

	Up to 69 Years of Age		70 Years and Over		*χ* ^2^
	*n*	*%*	*n*	*%*	
Gym	144	73.5	52	26.5	14.146 **
Dance	28	51.9	26	48.1	
Water activities	37	74.0	13	26.0	
Yoga/Pilates	32	55.2	26	44.8	

Note: *n* = number of participants; % = percentage; * *N* = 358; ** *p* < 0.05.

## Data Availability

The database is under the custody of the IP with anonymized data.
